# Air Pollution and Diabetes Risk: Assessing the Evidence to Date

**DOI:** 10.1289/ehp.123-A134

**Published:** 2015-05-01

**Authors:** Wendee Nicole

**Affiliations:** Wendee Nicole was awarded the inaugural Mongabay Prize for Environmental Reporting in 2013. She writes for *Discover*, *Scientific American*, *National Wildlife*, and other magazines.

Many studies have reported associations between ambient air pollution and cardiovascular disease, asthma, and cancer.^1^ Diabetes mellitus also is a risk factor for vascular and respiratory diseases, and development of these outcomes in people with diabetes may be exacerbated by exposure to air pollution.^2^ In this issue of *EHP*, a team of European scientists conducted a systematic review to evaluate whether air pollution exposure is also associated with developing diabetes itself.^3^

The researchers systematically searched databases for English-language articles addressing diabetes and outdoor air pollution in human subjects. They screened 636 studies and identified 13 that addressed the research question of interest. Eight pertained to type 2 diabetes, two pertained to type 1 diabetes, and three pertained to gestational diabetes. Seven of the studies on type 2 diabetes—selected because they reported air particle concentrations the same way—were pooled in a meta-analysis.

**Figure:**
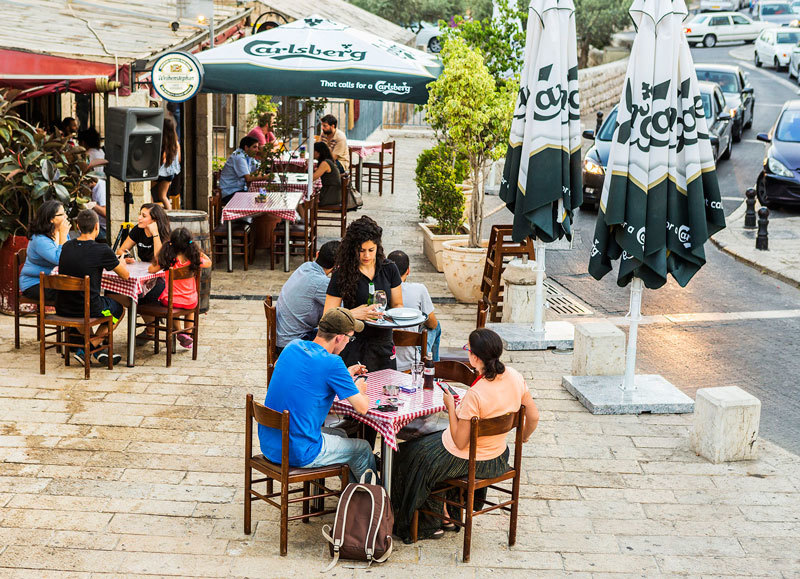
Subclinical inflammation, which can be caused by exposure to particulate matter, is “a major driving force” behind diabetes. © Atlantide Phototravel/Corbis

Based on three available longitudinal studies on exposure to fine particulate matter (PM_2.5_), the authors estimated a 10% increased risk of type 2 diabetes per 10-mg/m^3^ increase in exposure. For nitrogen dioxide (NO_2_), there were two longitudinal and two cross-sectional studies available, which suggested an 8% increase in type 2 diabetes per 10-mg/m^3^ increase in exposure.^3^

For both NO_2_ and PM_2.5_, estimated effects were more pronounced in females than males.^3^ “This was one of the surprising findings of our study, considering that men are usually at higher risk for type 2 diabetes,” says coauthor Ikenna Eze, a PhD candidate at the Swiss Tropical and Public Health Institute. “There could also be some unexplained sex-based physiologic differences which could account for this.” Alternatively, women generally tend to stay around the home more than men,^4^ hence residence-based exposure estimates may have better captured their actual exposures.

Positive associations reported in the epidemiologic literature give credence to the hypothesis that air pollution exposure may increase the risk of developing diabetes, says Patricia Coogan, an epidemiology research professor at Boston University and coauthor of one of the studies reviewed.^5^ “Even more convincing, I think, are the animal and clinical studies indicating that air pollution can affect insulin sensitivity and other biologic pathways relevant to diabetes,” Coogan says.

Ursula Krämer, a professor at the IUF-Leibniz Research Institute for Environmental Medicine whose study was included in the meta-analysis,^6^ believes the association between air pollution exposure and development of diabetes is plausible. “Subclinical inflammation is a major driving force for the incidence of diabetes, and particle pollution can cause subclinical inflammation,” she says. “I fully agree with the main conclusion of the authors: Research should be expanded to developing countries, where a steep increase in diabetes type 2 was observed in the last decade and where outdoor and indoor pollution is much higher than in Europe and North America.”
